# Novel controlled and targeted releasing hydrogen sulfide system exerts combinational cerebral and myocardial protection after cardiac arrest

**DOI:** 10.1186/s12951-021-00784-w

**Published:** 2021-02-06

**Authors:** Xiaotian Sun, Yiqing Wang, Shuyan Wen, Kai Huang, Jiechun Huang, Xianglin Chu, Fangrui Wang, Liewen Pang

**Affiliations:** Department of Cardiothoracic Surgery, Huashan Hospital of Fudan University, 12th Wulumuqi Rd, 200040 Shanghai, China

**Keywords:** Cardiac arrest, Hydrogen sulfide, Mesoporous iron oxide nanoparticles, Ischemia and reperfusion injury, Combinational cerebral and myocardial protection

## Abstract

**Background:**

Cardiac arrest (CA) is a leading cause of death worldwide. Even after successful cardiopulmonary resuscitation (CPR), the majorities of survivals are companied with permanent myocardial and cerebral injury. Hydrogen sulfide (H_2_S) has been recognized as a novel gasotransmitter exerting multiple organ protection; however, the lacks of ideal H_2_S donors which can controlled release H_2_S to targeted organs such as heart and brain limits its application.

**Results:**

This work utilized mesoporous iron oxide nanoparticle (MION) as the carriers of diallyl trisulfide (DATS), with polyethylene glycol (PEG) and lactoferrin (LF) modified to MIONs to acquire the prolonged circulation time and brain-targeting effects, and a novel targeted H_2_S releasing system was constructed (DATS@MION-PEG-LF), which exhibited excellent biocompatibility, controlled-releasing H_2_S pattern, heart and brain targeting features, and the ability to be non-invasive traced by magnetic resonance imaging. DATS@MION-PEG-LF presented potent protective effects against cerebral and cardiac ischemic injury after CA in both *in vitro* hypoxia/reoxygenation models and *in vivo* CA/CPR models, which mainly involves anti-apoptosis, anti-inflammatory and anti-oxidant mechanisms. Accordingly, the cardiac and cerebral functions were obviously improved after CA/CPR, with potentially improved survival.

**Conclusions:**

The present work provides a unique platform for targeted controlled release of H_2_S based on MIONs, and offers a new method for combinational myocardial and cerebral protection from ischemic injury, bringing considerable benefits for CA patients.
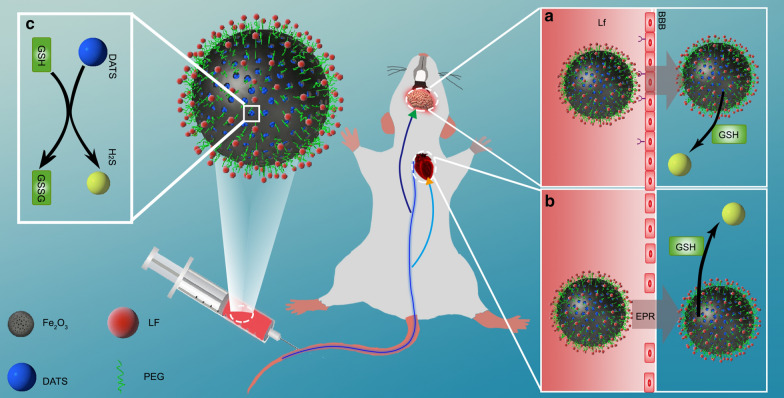

## Background

Cardiac arrest (CA) claims around 450,000 lives per year in the United States, and only 10% of which can survive to hospital discharge [1, 2]. Even after successful cardiopulmonary resuscitation (CPR) and return of spontaneous circulation (ROSC) following CA, the majorities of survival patients are companied with multiple organ lesions [1, 3]. Heart and brain are recognized as the two organs most sensitive to ischemic and reperfusion injury (I/R), and only 5 ~ 17 % of survivors can avoid long term myocardial and cerebral injury [3]. Therapeutic hypothermia is the most applied treatment for post-resuscitation care after CA/CPR, however, it is also associated with severe adverse effects including hyperglycemia, arrhythmia, and infection [4]. Therefore, novel approaches for protecting patients from myocardial and cerebral injuries after CA is still in highly demand.

Recently, hydrogen sulfide (H_2_S) has been reported as a novel gasotransmitter that can exert various physiological effects, particularly in the cardiovascular system and center nervous system (CNS). Increasing evidences suggest that the administration of exogenous H_2_S can successfully attenuate myocardial and cerebral I/R injuries, involving various mechanisms including anti-inflammation, anti-apoptotic, and antioxidant [5, 6]. However, the lack of ideal H_2_S donors that can be targeted to heart and brain organs and controlled release H_2_S largely limits the application of H_2_S in combinational cerebral and myocardial protection. Sodium hydrosulphide (NaHS) is the most commonly used H_2_S donor in animal experiments [7, 8], but the instant release of H_2_S from NaHS cannot mimic the slow and continuous H_2_S generation in the *in vivo* environment, which also leads to imprecise experimental results and detrimental effects [8, 9]. Some slow releasing H_2_S donors such as morpholin-4-ium 4-methoxyphenyl-morpholino-phosphinodithioate (GYY4137) can increase H_2_S levels slowly, but their release processes are hardly to be regulated [9], and cannot be targeted to certain organs such as brain and heart, limiting the therapeutic efficiency in lesion organs. In the previous study, we have utilized the mesoporous silica nanoparticles (MSN) to load the H_2_S donor diallyl trisulfide (DATS), and a GSH-triggered, slow releasing H_2_S system has been constructed (DATS-MSN), which can slowly release H_2_S *in vitro* and *in vivo* [10]. However, MSN cannot be utilized by mammal [11], leading to some biosafety concerns. Also, MSN cannot be traced by magnetic resonance (MRI), which is not suitable for a drug delivery system. Furthermore, MSN are easily adsorbed by plasma protein with limited circulation time, and not able to transport across blood-brain barrier (BBB), limiting the effects of its loading drugs in central nervous system (CNS).

**Fig. 1:**
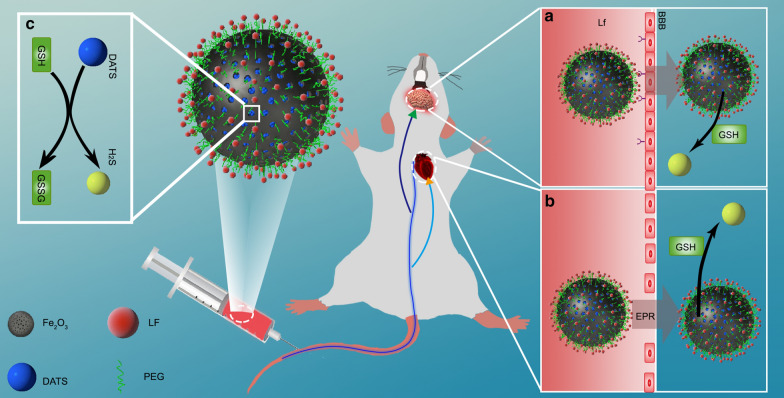
The schematic of design and working principle of the novel H_2_S targeted releasing system DATS@MION-PEG-LF

Herein, we have constructed a novel H_2_S releasing system utilizing mesoporous iron oxide nanoparticle (MION) as the carriers of DATS. Unlike MSN, MION could be fully metabolized and easily traced *in vivo*, and the hydroxyl surface of which also makes it easily modified with functional groups. Polyethylene glycol (PEG) was modified to MIONs to acquire the prolonged circulation time, and lactoferrin (LF) was introduced to help nanoparticles to across the BBB and gain brain-targeting effects. Then a biocompatible, trackable, heart and brain targeted, controlled-releasing H_2_S system was developed (DATS@MION-PEG-LF) (Fig. 1). Biocompatibility, H_2_S release pattern, organ targeting ability, and the myocardial and cerebral protective effects were investigated both *in vitro* and *in vivo*.

## Results

### Characterization

**Fig. 2:**
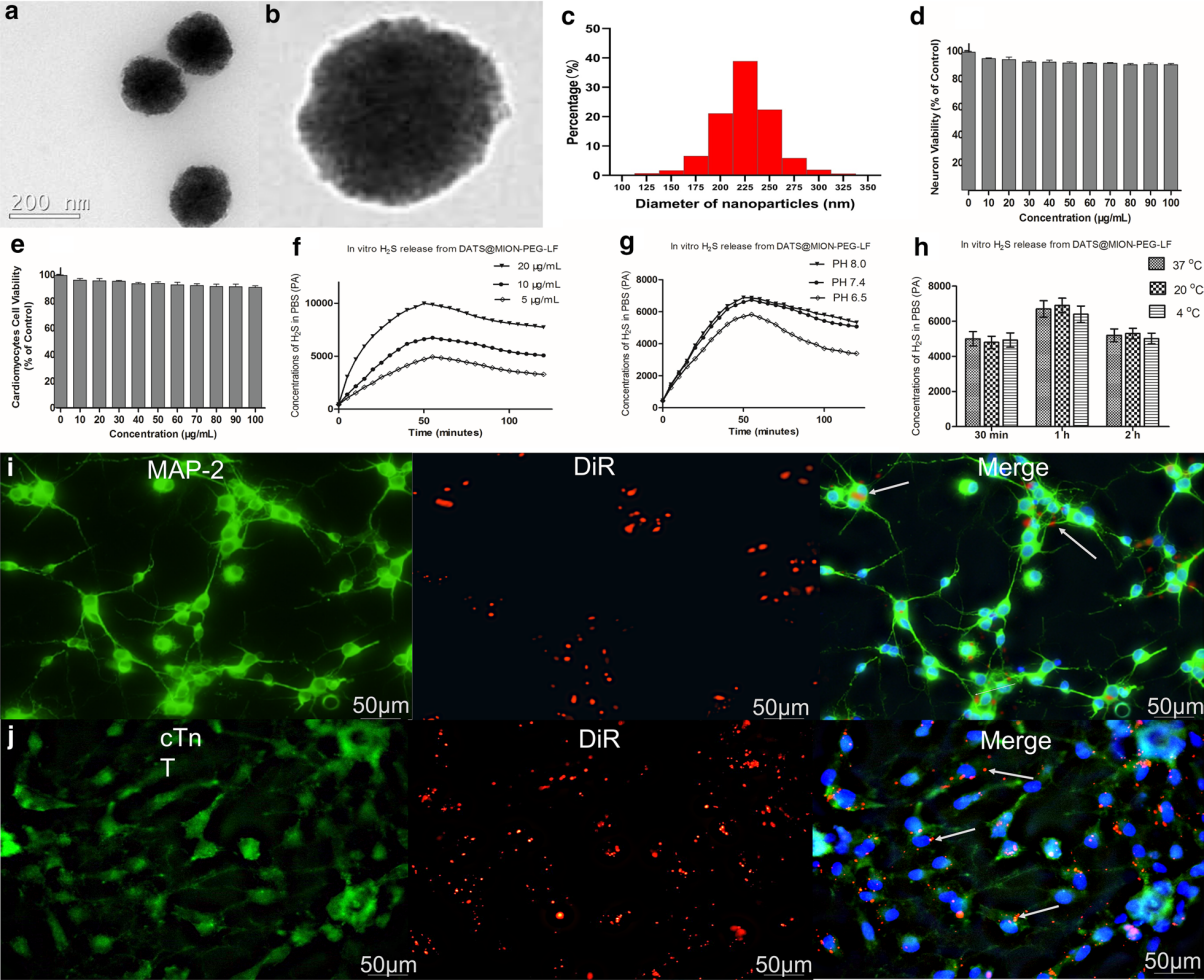
Characterization, *In vitro* Cytotoxicity Assays, H_2_S Release Assessment, and Cellular Uptake of DATS@MION-PEG-LF. **a** TEM and **b** enlarged TEM images. **c** The DLS results demonstrated a narrow size distribution of the nanoparticles. **d** Neuron and **e** cardiomyocytes cell viability after incubated for 24 h in different concentrations of DATS@MION-PEG-LF (mean ± SEM, n = 3). **f** Release of H_2_S from DATS@MION-PEG-LF in PBS solution. DATS@MION-PEG-LF (20, 10, and 5 µg/mL) with GSH (2 mM) (pH 7.4, 37 ^o^C). **g** DATS@MION-PEG-LF (10 µg/mL) with GSH (2 mM) at pH 7.4, 6.5, and 8.0 (37 ^o^C). **h** DATS@MION-PEG-LF (10 µg/mL) with GSH (2 mM) at 37, 20, and 4 ^o^C (pH 7.4). Fluoresce microscopic images after 4 h incubation with DiR@MION-PEG-LF (50 µg/mL) in neurons (**i**) and cardiomyocytes (**j**). Red: DiR@MION-PEG-LF, Green: cytoplasm stained by cTnT (cardiomyocytes) and MAP-2 (neurons), Blue: nucleus stained by DAPI

TEM images indicate that MIONs are monodisperse with uniform size and regular mesoporous (Fig. 2a, b). The nanoparticles had a narrow size distribution around 229 ± 32 nm by DLS assay (Fig. 2c). The conjugation of Mal-PEG-NHS and LF to MIONs were confirmed by FT-IR spectroscopy (Additional file 1: Figure S2). According to the standard curve given by the ELISA kit, the average amounts of LF conjugated onto MION were around 82 per MION. After drug loading, the average weight of the DATS loaded nanoparticles was founded to be around 1.64 times that of free nanoparticles, calculating the drug loading efficiency of MION-PEG-LF equal to 39 %.

### In vitro cytotoxicity assays, H_2_S Release Assessment and cellular uptake

DATS@MION-PEG-LF showed no significant toxicity (cell viability > 90 %) at the concentration up to 100 µg/mL in both cardiomyocytes and neurons (Fig. 2d, e), suggesting that it can be safely used as H_2_S donor *in vitro* in this range of concentrations. DATS@MION-PEG-LF released moderate amounts of H_2_S in PBS steadily, which varied with the concentration DATS@MION-PEG-LF (Fig. 2f). The release of H_2_S from DATS-MSN is pH dependent (Fig. 2g) as it is significantly inhibited in an acidic (pH 6.5) compared with neutral (pH 7.4) environment, while alkaline PBS (pH 8.0) did not apparently affect H_2_S release. Temperatures (37, 20 and 4 °C) have negligible impacts on generation and release of H_2_S (Fig. 2h). H_2_S formation was observed at the beginning of the drug adding, and gradually increased over the first hour until reaching a plateau, indicating a steady and sustained H_2_S release feature for DATS@MION-PEG-LF. Red fluorescence of DiR@MION-PEG-LF was observed in cytoplasm, proving that the nanoparticles are phagocytized by neurons and cardiomyocytes successfully (Fig. 2i, j). The fluorescent intensity of the nanoparticles is proportional to their number concentration, and a moderate concentration in cardiomyocytes was observed.

### Protection effects of DATS@MION-PEG-LF against Hypoxia/Reoxygenation induced damage of cardiomyocytes and neurons

**Fig. 3:**
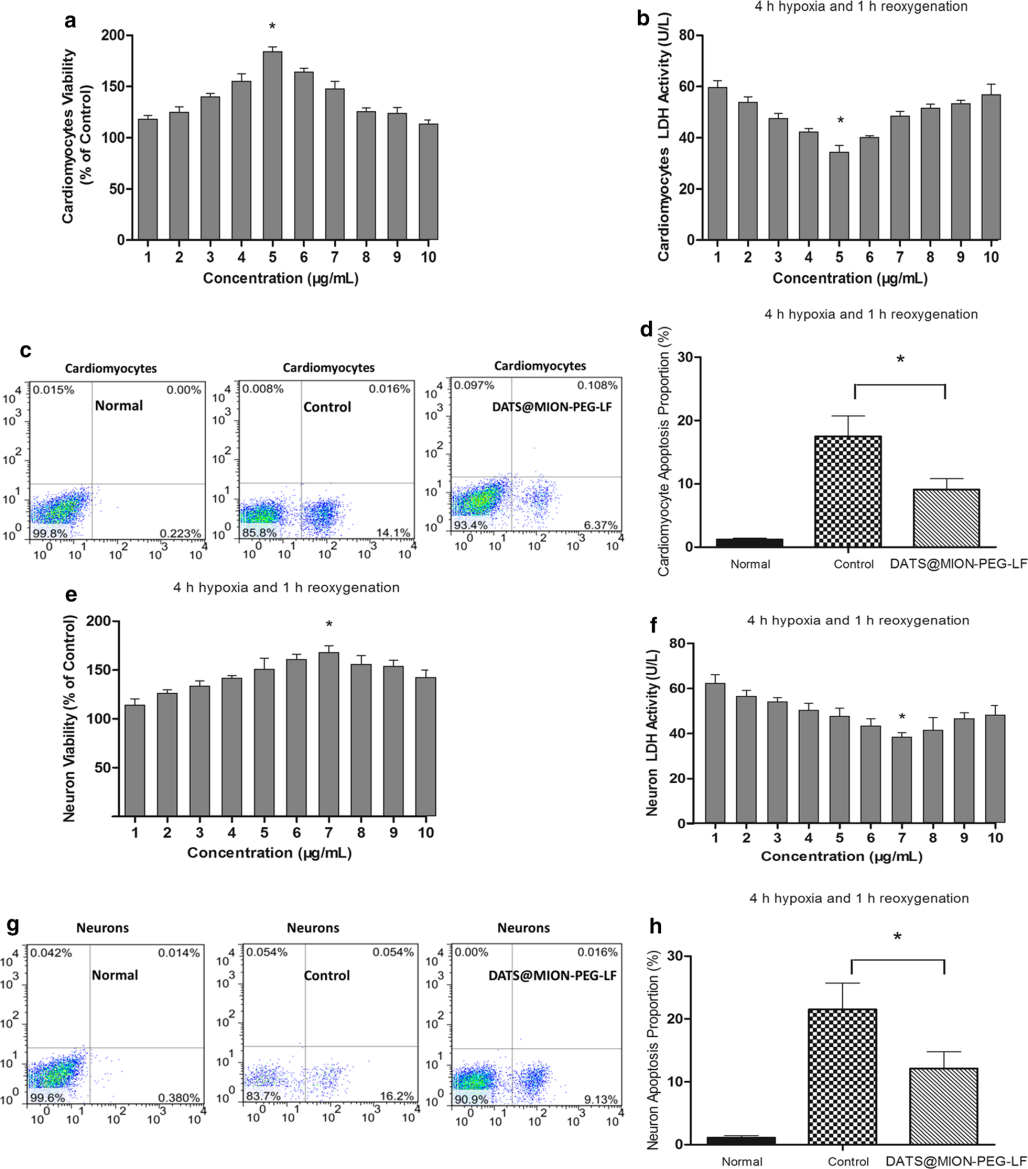
*In vitro* protective effects of DATS@MION-PEG-LF against hypoxia/reoxygenation induced damage in rat cardiomyocytes and neurons. Cardiomyocytes cell viability (**a**), lactate dehydrogenase (LDH) activity (**b**), flow cytometry assay (**c**), and apoptosis proportion (**d**) were evaluated after 4 h hypoxia and 1 h reoxygenation procedure; neuron cell viability (**e**), LDH activity (**f**), flow cytometry assay (**g**), and apoptosis proportion (**h**) were evaluated after same experimental protocols. Flow cytometry assay and apoptosis proportion evaluation were performed at the most protective concentration (5 µg/mL for cardiomyocytes, and 7 µg/mL for neurons). *: P < 0.05 compared with the Control (mean ± SEM, n = 3)

In cardiomyocytes cell viability assay, obvious protective effects of DATS@MION-PEG-LF against hypoxic damage was observed at the concentrations among 0 ~ 10 µg/mL, and the most effective concentration was around 5 µg/mL (Fig. 3a). The LDH evaluation showed similar results, which was significantly decreased by DATS@MION-PEG-LF administration (Fig. 3b). Meanwhile, DATS@MION-PEG-LF (5 µg/mL) can significantly decrease the apoptosis proportion compared with the Control group by flow cytometry assay (Fig. 3c, d). *In vitro* neurons experiments showed similar results, with increased cell viability (Fig. 3e), decreased LDH levels (Fig. 3f), and reduced apoptosis proportion (Fig. 3g, h), and the most effective concentration was around 7 µg/mL.

### Biodegradation and in vivo safety Assessment

**Fig. 4:**
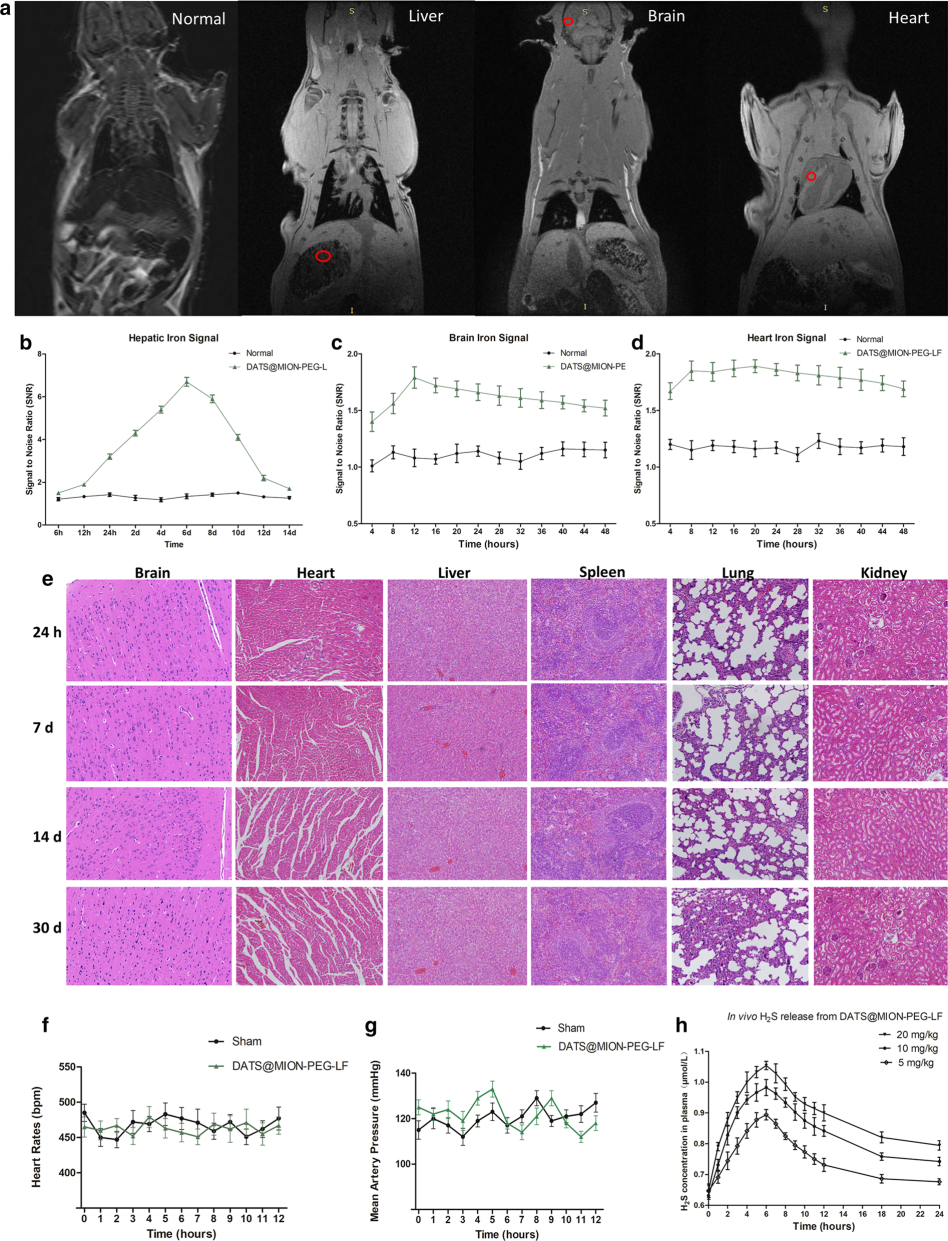
Biodegradation and *In vivo* Safety Assessment of DATS@MION-PEG-LF. **a** Coronal view of mouse by MRI scan, the hepatic, brain, and heart religion were selected for detecting fluorescence intensity (red circle). **b** Time course assessment of DATS@MION-PEG-LF intensity in mice by fluorescence intensity assessment, compared with the untreated mice (Normal). **c** Toxicity assessment of DATS@MION-PEG-LF via H&E staining in vital organs. **d** Heart rates and **e** mean blood pressure in 12 h after injection. **f** H_2_S concentration in plasma after injection of DATS@MION-PEG-LF at different doses. (mean ± SEM, n = 6)

It has been reported that ferric oxide nanoparticles are mainly accumulated and metabolized in liver and spleen organs [12, 13]. Accordingly, the present work studied the liver accumulation of DATS@MION-PEG-LF by MRI to present its biodegradation process: the hepatic fluorescence intensity reached its peak value around the 6th day after the injection, and then declined over the next 8 days, implying the gradual degradation and clearance of the nanoparticles (Fig. 4a, b). Meanwhile, the organ distribution assessment demonstrated that the brain and heart fluorescence intensities were elevated since 4 h after injection, and stayed increased within the 48 h experiments (Fig. 4a, c, and d). H&E staining at 2, 24 h, 7 and 30 days all revealed great biocompatibility of DATS@MION-PEG-LF: DATS@MION-PEG-LF did not result in obvious damage to any vital organs after drug administration by H&E (Fig. 4e). Meanwhile, compared with the Sham, DATS@MION-PEG-LF made no influence to either heart rates or mean artery pressure within 12 h (Fig. 4f, g), suggesting no obvious harm to hemodynamics. The Hematological and serological examinations at 2, 24 h and 7 days after injection of DATS@MION-PEG-LF also proved the *in vivo* biosafety of DATS@MION-PEG-LF (Additional file 1: Table S1). As shown in Fig. 4h, H_2_S release from DATS@MION-PEG-LF in plasma is slow and stable, which obviously increased the plasma H_2_S levels from the first time point, peaked at the 6th hour, and remained elevated over the 24 h experiments. The releasing of H_2_S in plasma was also related to the dose of DATS@MION-PEG-LF administrated. The pharmacokinetic parameters of different doses DATS@MION-PEG-LF were described in Additional file 1: Table S2.

### In vivo targeting ability of DATS@MION-PEG-LF to Heart and brain

**Fig. 5:**
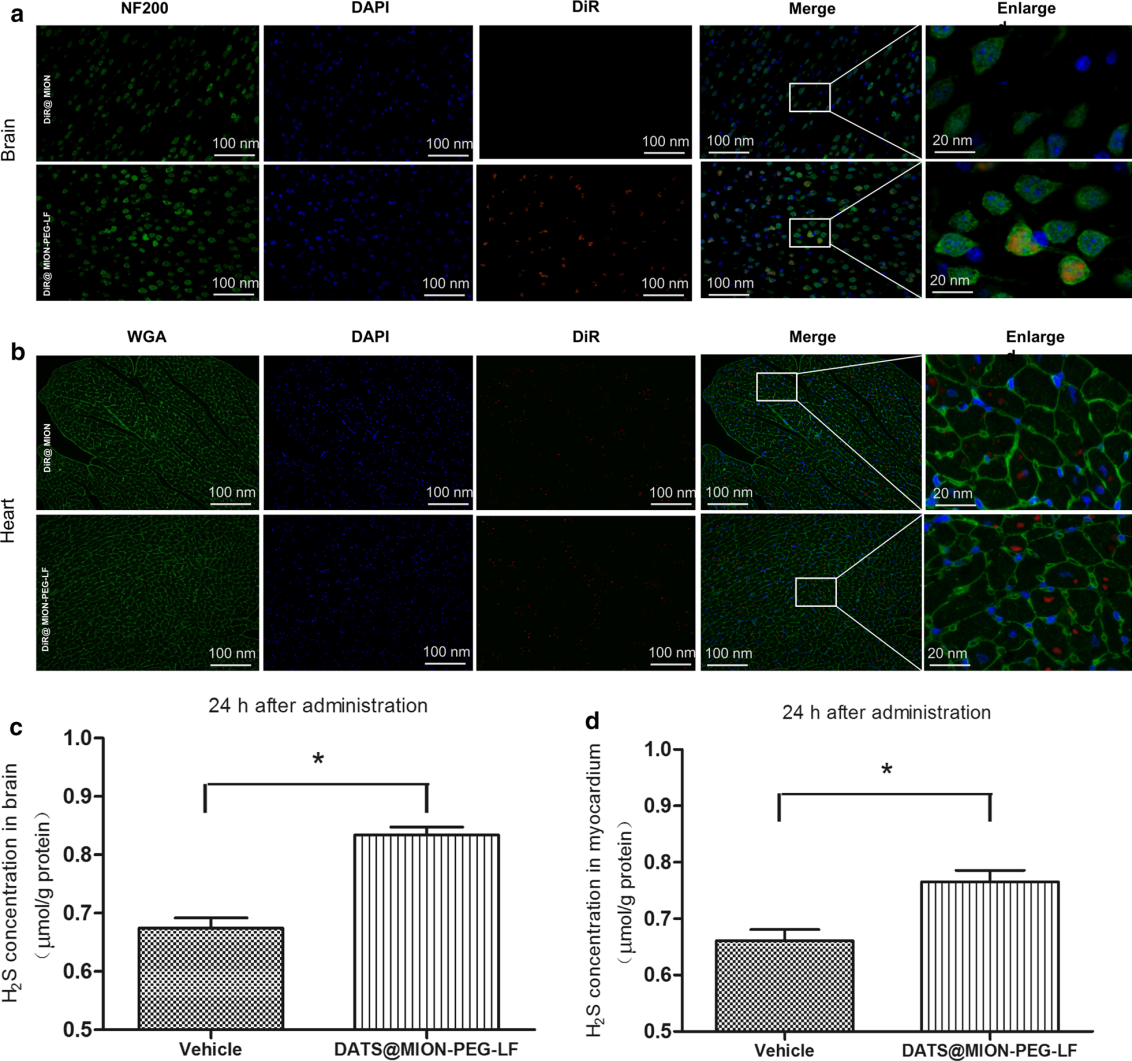
Immunofluorescence images of frozen brain (**a**) and heart tissues (**b**) at 24 h after injection of DiR@MION and DiR@MION-PEG-LF were evaluated. The DAPI-stained nuclei gave out blue fluorescence under excitation and DiR-loaded nanoparticles showed red fluorescence under excitation. H_2_S concentrations were measured in brain tissues (**c**) and myocardium (**d**) at 24 h after administration of DATS@MION-PEG-LF. *P < 0.05 compared with the Vehicle group (mean ± SEM, n = 6)

*Ex vivo* imaging experiments presented that the DiR was successfully loaded into MION and MION-PEG-LF to form DiR@MION and DiR@MION-PEG-LF, bearing the ability to represent the distributions of these nanocarriers in the *in vivo* experiments (Additional file 1: Figure S3). At 24 h after *i.v* injection of DiR@ MION-PEG-LF, the brain tissue slices showed the obvious accumulation of DATS@MION-PEG-LF, which was mainly localized in neurons, this was not observed in the DiR@MION group (Fig. 5a), indicating the good targeting ability of DATS@MION-PEG-LF to brain via LF modification. At the same time point, the heart tissue slices of DiR@ MION-PEG-LF and DiR@ MION groups both showed scattered DiR fluorescent signals dispersed in cardiomyocytes, indicating that the entrance of the nanoparticles to the heart tissues could be via the other mechanisms such as enhanced permeability and retention effect (EPR) effects (Fig. 5b). Accordingly, the H_2_S content in tissues of the heart (Fig. 5c) and brain (Fig. 5d) were also elevated compared with the Vehicle group at 24 h after the DATS@MION-PEG-LF administration, proving its delivery ability of H_2_S to the heart and brain organs.

### DATS@MION-PEG-LF protects ischemic brain after CA/CPR

**Fig. 6:**
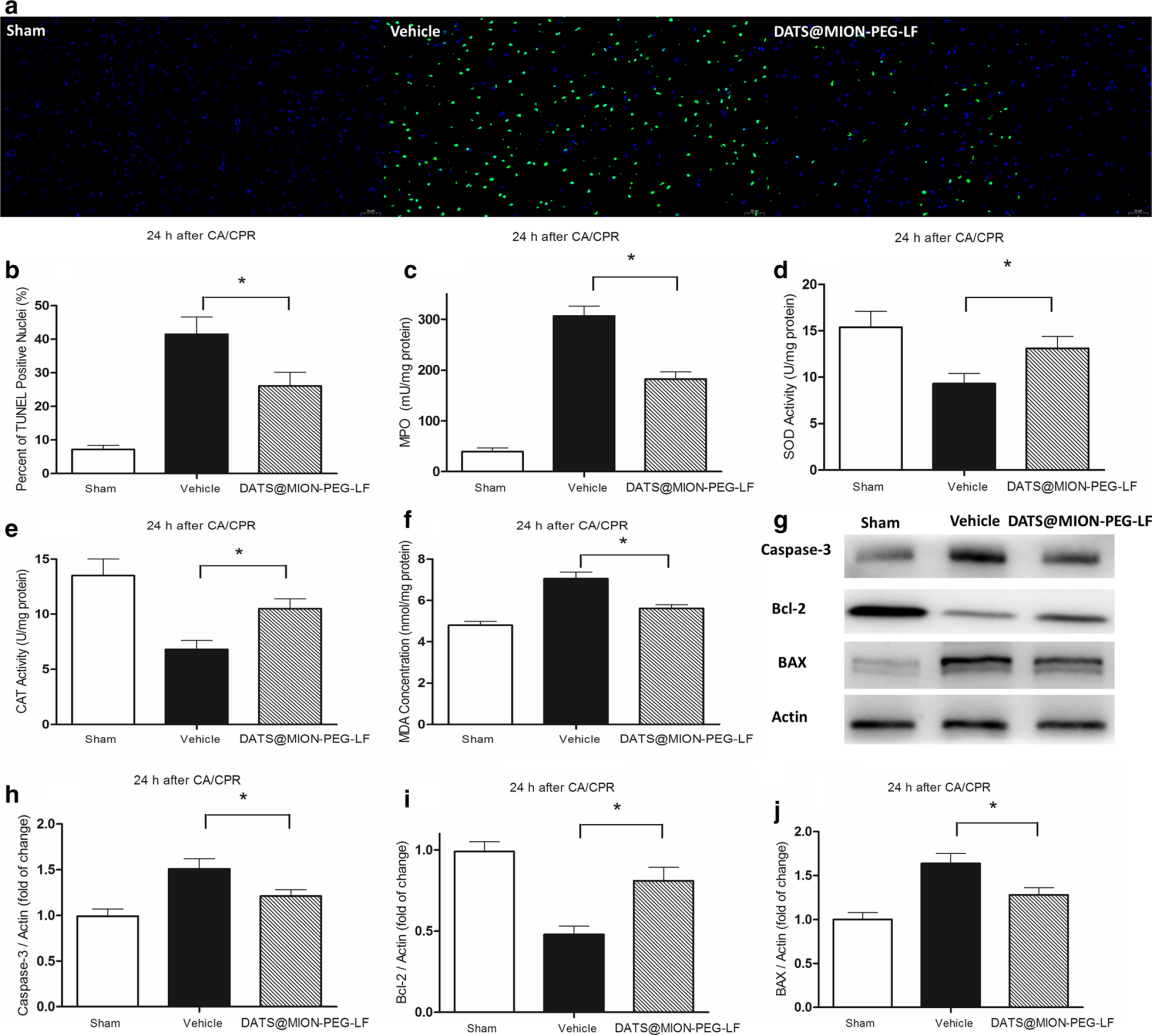
DATS@MION-PEG-LF protects ischemic brain after CA/CPR. TUNEL staining detected neurons apoptosis at 24 h after CA/CPR. Nuclei with green staining indicate TUNEL positive cells (200×) (**a**); the percentage of TUNEL positive cells to total neurons in individual groups (**b**); myeloperoxidase (MPO) activities (**c**), superoxide dismutase (SOD) activities (**d**), catalase (CAT) activities (**e**), and levels of malonydialdehyde (MDA) contents (**f**) were measured in brain tissue at 24 h after CA/CPR. The levels of Capase-3, Bcl-2, and Bax in brain tissues were tested using western blot assay (**g**); the intensity of each band was quantified by densitometry, and data were normalized to the Actin signal (**h–j**). *P < 0.05 compared with the Vehicle Group (mean ± SEM, n = 6)

As shown in Fig. 6a, DATS@MION-PEG-LF protected against brain apoptosis following 24 h of reperfusion. The apoptosis index was significantly decreased in the novel H_2_S donor group compared with the Vehicle group (Fig. 6b). The rats receiving the novel H_2_S donor also displayed a reduced degree of cerebral inflammation in comparison with the Vehicle group, with a reduction in MPO activities (Fig. 6c). DATS@MION-PEG-LF also resisted oxidative stress in brain tissues following the CA/CPR protocol, displaying an effective preservation of SOD activity (Fig. 6d) and CAT activity (Fig. 6e), and a reduced levels of MDA (Fig. 6f) compared with the Vehicle group. As shown in Fig. 6g–j, the I/R injury induced the expression of Caspase-3 and BAX, and reduced the expression of Bcl-2 in the Vehicle group, and the DATS@MION-PEG-LF exhibited decreased levels of Caspase-3 and BAX, and increased levels of Bcl-2, respectively.

### DATS@MION-PEG-LF protect ischemic myocardium after CA/CPR

**Fig. 7:**
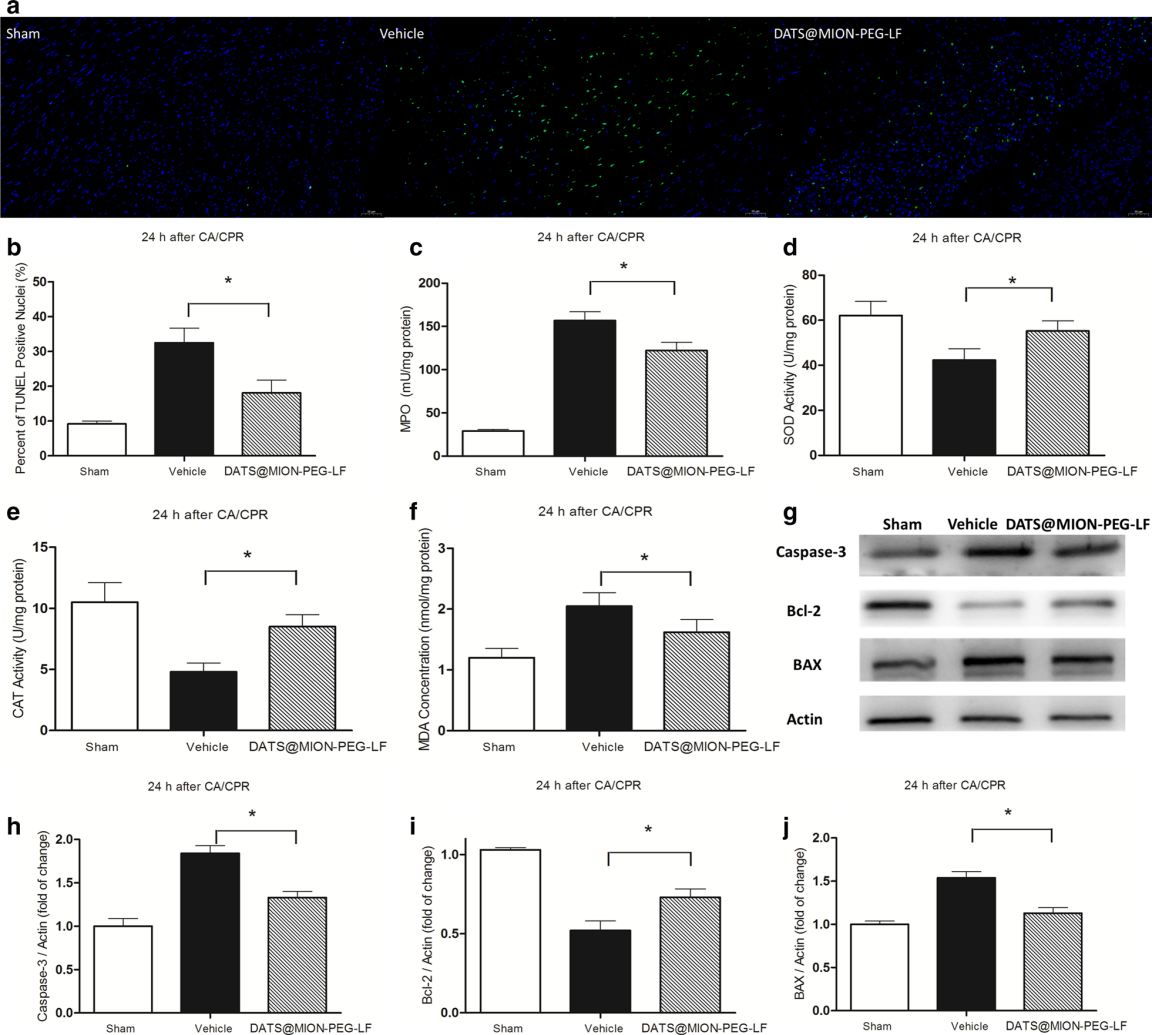
DATS@MION-PEG-LF protects ischemic heart after CA/CPR. TUNEL staining detected cardiomyocytes apoptosis at 24 h after CA/CPR. Nuclei with green staining indicate TUNEL positive cells (200×) (**a**); the percentage of TUNEL positive cells to total cardiomyocytes in individual groups (**b**); myeloperoxidase (MPO) activities (**c**), superoxide dismutase (SOD) activities (**d**), catalase (CAT) activities (**e**), and levels of malonydialdehyde (MDA) contents (**f**) were measured in heart tissue at 24 h after CA/CPR. The levels of Capase-3, Bcl-2, and Bax in myocardium were tested using western blot assay (**g**); the intensity of each band was quantified by densitometry, and data were normalized to the Actin signal (**h**–**j**). *: P < 0.05 compared with the Vehicle Group (mean ± SEM, n = 6)

The protection effects of DATS@MION-PEG-LF to ischemic myocardium after CA/CPR protocol were similar to that of ischemic brain. By analyzing the ischemic myocardial tissue at 24 h after reperfusion, DATS@MION-PEG-LF group showed significantly protection effects compared with Vehicle group, with obviously inhibited myocardial apoptosis (Fig. 7a, b), reduced myocardial neutrophil accumulation (Fig. 7c), resisted myocardial oxidative stress (Fig. 7d–f), and positive regulation of apoptosis related proteins (Fig. 7g–j).

### DATS@MION-PEG-LF preserved cerebral and cardiac function after CA/CPR

**Fig. 8:**
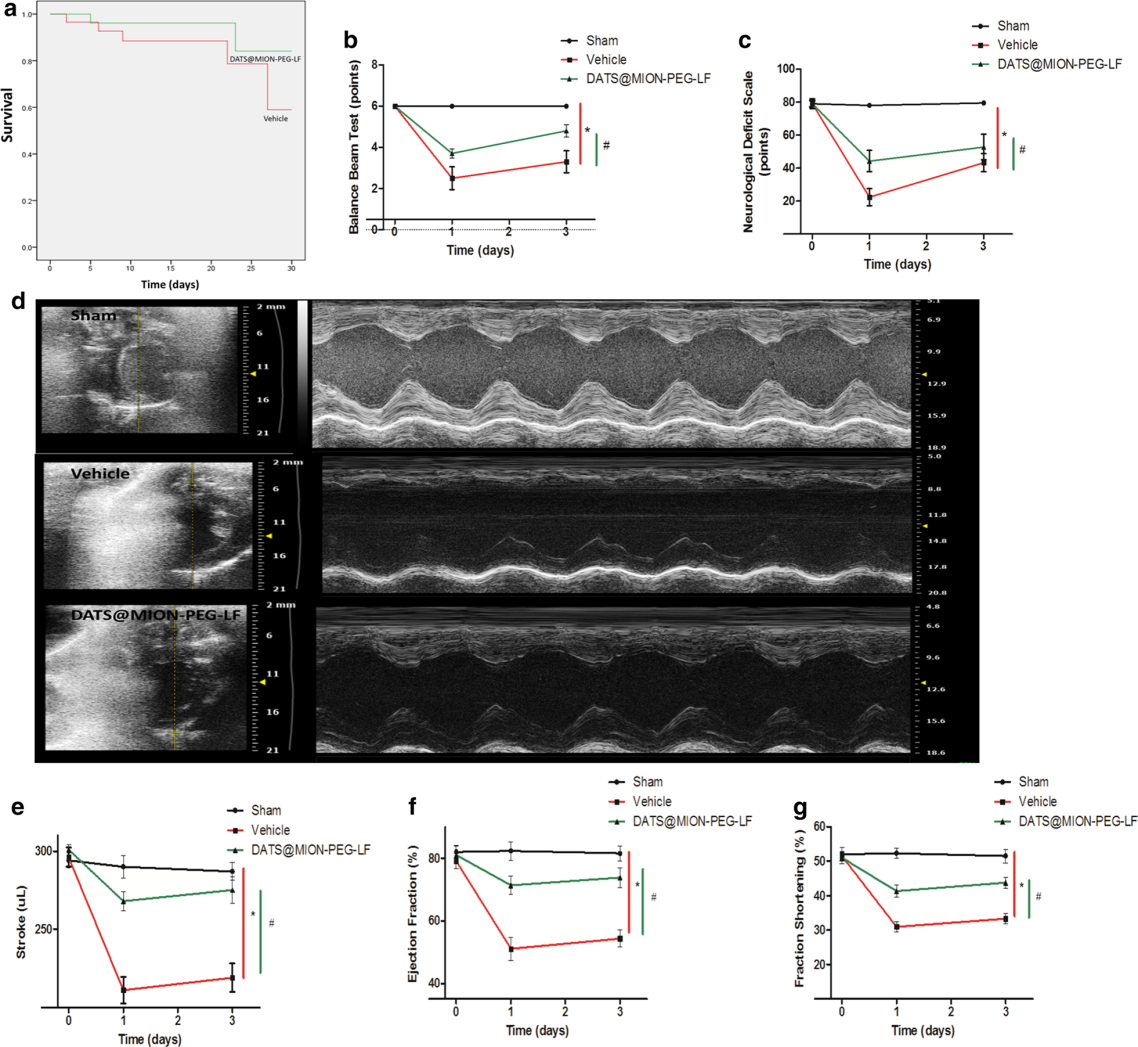
Cardiac and cerebral function evaluation after CA/CPR. Survival estimates by Kaplan–Meier in Vehicle and DATS@MION-PEG-LF groups within 30 d after CA/CPR (n = 30) (**a**); balance beam test scores (**b**) and neurological deficit scale scores (Emulsifying activity (OD_500_) and emulsion index IE24c) in Sham, Vehicle and DATS@MION-PEG-LF groups at 1 and 3 days after CA/CPR; representative M-mode images from individual groups evaluated at 3 days after CA/CPR (**d**); stroke volume (**e**), ejection fraction (**f**), and fractional shortening (**g**) measured by M-mode echocardiography at 1 d and 3 d after CA/CPR. *P < 0.05 compared between the Sham and the Vehicle Groups; #P < 0.05 compared between the DATS@MION-PEG-LF and the Vehicle Groups (mean ± SEM, n = 6)

As shown in Fig. 8a, the survival rates had a tendency to be improved by DATS@MION-PEG-LF administration compared with the Vehicle. Correspondingly, the balance beam test scores and NDS scores were also obviously increased in the DATS@MION-PEG-LF group at 1 and 3 days after CA/CPR (Fig. 8b, c). Echocardiography results (Fig. 8d) revealed that DATS@MION-PEG-LF effectively preserved the cardiac function at 1 and 3 days after the CA/CPR, presenting an increased stroke volume (Fig. 8e), ejection fraction (Fig. 8f) and fractional shortening (Fig. 8g) when compared with the Vehicle group.

## Discussion

The present treatment for early ischemic injury of heart and brain after CA is still in the challenge. As the main therapy strategy currently, therapeutic hypothermia causes increased risks of pneumonia and sepsis, and is far from sufficiency to improve the outcomes [14]. Therefore, it still calls for a convenient and efficient treatment method to combinational protect heart and brain organs, which can be easily applied at the early stage after CA. Bearing various physiology effects including anti-inflammatory, anti-apoptosis and anti-oxidant effects of H_2_S, a targeted and slow releasing H_2_S system emerges as a promising tool for cerebral and myocardial protection from ischemic injury. The novel system presented good biocompatibility, controlled-releasing pattern, specific organ targeting ability, and combinational heart and brain protective effects.

MIONs were utilized as the framework of the new H_2_S releasing system for many specific characteristics. Firstly, MIONs present superior biocompatibility with no obvious *in vivo* toxicity. Physiological iron can be found in various tissues in forms of hemosiderin, ferritin and transferrin, and MIONs could be transformed into ferritin or move into the intracellular storage iron pool with the similar homeostasis as physiological iron [15]. It has been widely reported that the MIONs were biodegraded mainly by liver, and excreted through urine and feces by the hepatobiliary process and urinary pathways; the eventual excretion could last for 21 days [15]. The present study demonstrated the superior biosafety of MIONs based H_2_S releasing system both in *in vitro* and *in vivo* experiments, with excellent cell viability and no signs of organ damage. Secondly, bearing excellent superparamagnetism, MION enable itself to be traced non-invasively *in vivo* through MRI techniques, which is extremely important in pharmacokinetics and pathological diagnosis studies [16]. Our study utilized the traceable feature of MIONs to demonstrate its biodegradation process *in vivo*. Finally, the hydroxyls surface of MION endows itself the ability to be easily modified by various ligands [17], which was utilized to introduce PEG and LF in this study.

To guarantee the therapy efficiency, an ideal drug donor should bear a long circulation time with slow-releasing pattern. The diameter of MIONs is around 200 nm, which could be slowly degraded and cleared by liver with relatively long circulation time [12]. Also, PEG modified MIONs could avoid the recognition by macrophages and removal by reticuloendothelial system, contributing prolonged duration in the plasma and increased organ uptake [18]. The slow-releasing mechanism of the novel H_2_S donor involves DATS molecules gradually escaping from the mesopores of nanoparticles to slow down their reaction with GSH molecules and slowly generate H_2_S [10]; the large surface area and pore volume of MIONs guaranteed this function. Therefore, the study provides a unique MION-based platform for long-term and controlled-release of H_2_S *in vitro* and *in vivo*.

In order to achieve transportation ability to across BBB, brain-targeting ligand LF was modified to MIONs. Consisting of tightly connecting endothelial cells, the BBB is the most important shield of CNS, but also a great obstacle for drug targeting delivery in cerebral injury. To overcome this, many receptor-mediated drug delivery systems are developed recently by coupling of vectors with specific receptors on the BBB to drug loading vehicles. LF is a mammalian cationic iron-binding glycoprotein belonging to the transferrin family, and LF receptor is found on the BBB of different species and transports LF across the BBB *in vitro* and *in vivo*. Two classes of binding sites for LF were found on cell membrane: a high affinity 105 kDa receptor protein and the low affinity glycosaminoglycans binding sites [19, 20]. It is reported that LF-modified DNA-loaded nanoparticles can transport across BBB through the receptor-mediated mechanism [19]. It is also reported that PEG-PLGA nanoparticles functionalized with LF can successfully appear in CNS [20]. Our study presented that only the LF-functionalized MIONs presented obvious fluorescent signals in brain, indicating adequate amounts of nanoparticles transported across into the brain tissues.

The uptake of modified MIONs by I/R myocardium is mainly depended on the enhanced permeability and retention (EPR) effects; in the myocardium undergoing I/R damage, the microvascular permeability would be increased and permit more nanoparticles to across into the cardiomyocytes; the degree of microvascular injury and increased permeability correlate with the severity of ischemia [21]. In the myocardial infarction (MI) models, the nanoparticle accumulation following intravenous administration could be observed in the infarcted myocardium at the acute and chronic phase of MI, respectively [22]. In our CA/CPR model, the MIONs based H_2_S releasing system was also successfully founded in ischemic myocardium.

As shown in this work, MIONs based drug delivery system present good biosafety in both *in vitro* and *in vivo* experiments. In the reported work, *i.v.* injection of LF connected nanoparticles may cause slight transient inflammatory reactions in the liver and spleen, but showed no obvious inflammatory reactions to other tissues such as brain and heart, with normal organ function and activities [19, 20]. Our developed nanoparticles also demonstrated excellent biocompatibility in brain and heart up to 30 d. However, further investigations should be concentrated on the long term toxicity on the important organs *in vivo*.

The accumulation of DATS@MION-PEG-LF in heart and brain is accompanied with elevated H_2_S content in related organs. For those previous H_2_S donors, the physiological effects of H_2_S is mainly depended on the plasma H_2_S supply. For H_2_S is easily transferred across respiratory membranes, the residence time in tissues is relatively short for fast-releasing H_2_S donors, somewhat limiting the biofunction effects of H_2_S in organs. A Nano-system which can be transported and stayed in the targeted organs could offer an accumulating H_2_S circumstance over a long period, and provide superior therapeutic functions. With considerable uptake by neurons and cardiomyocytes, DATS@MION-PEG-LF can exert superior corresponding physiological effects of H_2_S. As to the present study, DATS@MION-PEG-LF combinational protected ischemic brain and heart mainly via anti-apoptosis, anti-inflammatory, and anti-oxidant mechanisms. Many studies have reported that H_2_S can exert anti-apoptotic effects via the inactivation of caspases caused by I/R [23]. In the presented study, DATS@MION-PEG-LF administration was associated with H_2_S-mediated modulation through increasing the levels of Bcl-2, decreasing the levels of BAX, and reducing the activity of caspases-3. Accordingly, the cerebral and cardiac functions were obviously improved after CA/CPR, with potentially improved survival. Therefore, the present study provides a practical and safe way of H_2_S treatment in ischemic organs, and offers a new insight into combinational organ protection of a MION based targeted releasing H_2_S system.

## Conclusions

The present study reported a multiple organ targeted H_2_S releasing system based on MIONs, which exhibited good biocompatibility, controlled-releasing pattern, heart and brain targeting features, and ability to be non-invasive traced by MRI. DATS@MION-PEG-LF can present potent protective effects against cerebral and cardiac ischemic injury after CA in both *in vitro* cardiomyocytes and neuron hypoxia/reoxygenation models and *in vivo* CA/CPR models. It provides a unique safe platform for controlled release of H_2_S based on MIONs, and offers a new method for combinational myocardial and cerebral protection from I/R injury, bringing considerable benefits for CA patients.

## Materials and methods

### Materials and reagents

Newborn (6 g, 24 h) and adult male Sprague-Dawley rats (250–280 g, 8 w), as well as BALB/c-nu mice (15 g, 4 w) were used in this study. All animal experiments were approved by Institutional Animal Care and Use Committee of Department of Laboratory Animal Science of Fudan University (Grant No. 20160780A176), and confirmed with the Guide for the Care and Use of Laboratory Animals published by the US National Institutes of Health (NIH publication no. 85 − 23, revised 1996). DATS, 3-aminopropyltriethoxysilane (APTES), LF, N-succinimidyl-S-acetylthioacetate (SATA), and Mal-PEG-NHS were obtained from Sigma-Aldrich (St Louis, MO). Other reagents were of analytical grade and used as purchased. All the solutions were prepared by Milli-Q water and deaerated with high-purity nitrogen.

### Preparation of MIONs

The synthesis of MIONs followed our previously described protocol with minor modifications [24]. Briefly, styrene (9 mL), methacrylic acid (1 mL), and deionized water (70 mL) were mixed and dispersed. The suspension was then stirred and heated to 75 °C for 30 min. Then 10 mL of potassium peroxodisulfate (10 mg/mL) was gradually added and react for 24 h at 75 °C. The obtained polystyrene nanoparticles were mixed with 30 mL of ethylene glycol and 170 mL of deionized water. After that, 450 mg of ferrous chloride, 110 mg of potassium nitrate, and 2 g of methenamine were sequentially added and heated to 80 °C under nitrogen protection. After cooling and centrifuging, collected nanoparticles were washed with distilled water to remove residual reagents, and gradually heated to 500 °C for 3 h. After cooling to room temperature, MIONs were obtained.

### Synthesis of DATS@MION-PEG-LF

The surface modifications of MIONs consist of conjugating Mal-PEG-NHS with MION and conjugating LF with Mal-PEG-MION (Additional file 1: Figure S1). Firstly, 5 mL of APTES was hydrolyzed under catalyzation of HCl (pH 4.0) to form silane polymer with reactive saline bond, then 30 mL of deionized (DI) water and 10 mg of MIONs were added. The mixture was stirred for 4 h at 65 °C under nitrogen protection, and was eluted with ethanol and DI water. Then 20 mg of Mal-PEG-NHS (Mw: 5000) was added to the silane coated MIONs, and the mixture was allowed to react for 5 h at room temperature after 30 min nitrogen bubbling, following dialysis against DI water.

LF was activated before conjugated to Mal-PEG-MION according to previous method [25]. Briefly, LF and SATA were incubated in Hepes buffered saline (pH 7.0) at 1:8 molar ratios for 1 h at room temperature under constant shaking. Free SATA was removed by centrifugal ultrafiltration with the help of Millipore UFC801096 tubes (12,000 rpm, 30 min at 4 °C). SATA modified LF was reacted with 0.1 M hydroxylamine for 45 min at room temperature before coupling to maleimide then was incubated with Mal-PEG-MION for 2 h at room temperature. After incubation, MION-PEG-LF went through centrifugal ultrafiltration with a 30Kd cut-off tube to dislodge uncombined LF and other impurities. After that, MION-PEG-LF solutions underwent FT-IR spectroscopy assay to confirm the conjugation of Mal-PEG-NHS and LF to MIONs (Perkin Elmer Frontier FT-IR, spectra recorded from wavenumber 400–4000 cm^− 1^) (detailed in Additional file 1). The average amounts of LF conjugated to MION was estimated by enzyme linked immunosorbent assay (ELISA) kit method.

DATS was loaded into MION-PEG-LF based on the previous reported protocols [8, 10]. In brief, 1 mg of MION-PEG-LF and 1 mg of DATS were sequentially mixed in 5 mL of distilled water, followed by stirring for 9 h. Resident DATS were removed from the surface of MIONs by being washed with distilled water. Parts of cleaned DATS@MION-PEG-LF were freeze-dried under vacuum and weighed to count the loading efficiency of DATS (mass of drug loaded in nanoparticles/ mass of drug loaded nanoparticles × 100 %). Other parts of DATS@MION-PEG-LF were reserved in saline solutions at room temperature ready for use.

### Characterization

The structure of DATS@MION-PEG-LF was analyzed by high-resolution transmission electron microscopy (HRTEM) images which were recorded by a JEM-2100F (JEOL, Japan) and a Tecnai T20 (FEI, USA) transmission electron microscope (TEM). The diameters of samples were acquired by averaging size of 50 nanoparticles in TEM images. Dynamic light scattering (DLS) Autosizer 4700 (Malvern, MA, UK) was used to measure the size distribution. The conjugations of Mal-PEG-NHS/LF to MIONs were confirmed by FT-IR method (Additional file 1). The samples were tested at 25 °C with concentration of 5 µg/mL.

### Cytotoxicity assays of DATS@MION-PEG-LF

Cytotoxicity assay of DATS@MION-PEG-LF was assessed using primary neonatal cardiomyocytes and neurons according to the previously described method [10, 26]. The culture of cardiomyocytes and neurons were described in Additional file 1. The DATS@MION-PEG-LF was diluted with culture medium to gain a concentration range from 0 to 100 µg/mL, and was added to 96-well flat-bottomed tissue-culture plate with cell seeded. After incubation for 24 h, medium was removed, and cells were washed with PBS, then the medium was replaced with cell counting kit-8 (CCK-8) solution (Dojindo Laboratories, Kumamoto, Japan). The absorbance of individual wells was measured at 450 nm by a microplate reader (Molecular Devices, FlexStation 3, CA, USA). The results were expressed as the mean percentage of cell viability relative to control.

### In vitro cellular uptake

Fluorescent dye DiR was loaded to the MION-PEG-LF frameworks. The protocols of loading and the identification of DiR@MION-PEG-LF were described in Additional file 1. Neonatal rat cardiomyocytes and neurons were treated with the DiR@MION-PEG-LF (50 µg/mL) in 24-well plates for 4 h. After washed with PBS for thrice, the cells were fixed by 4 % paraformaldehyde for 15 min, penetrated by 0.1 % triton-X 100 for 20 min, stained by cTnT (cardiomyocytes) and MAP-2 (neuron) for 8 h and 4′, 6-diamidino-2-phenylindole (DAPI) for 15 min. Then the DiR@MION-PEG-LF (λex = 750 nm) inside cells were visualized using a fluoresce microscope (Olympus IX-71, Japan).

### In vitro H_2_S release of DATS@MION-PEG-LF

The *in vitro* release course of DATS@MION-PEG-LF was assessed by real time H_2_S-selective microelectrode assay: 5, 10, and 20 µg/mL of DATS@MION-PEG-LF was separately added to a glass chamber (World Precision Instruments, WPI, USA) containing PBS (100 mM, pH 7.4, 4 mL) with 2 mM of GSH at 37 °C. Then H_2_S formation was detected using ISO-H_2_S-2 sensor attached to an Apollo 1100 Free Radical Analyser (WPI, FL, USA). H_2_S release was real-time displayed by picoamps (PA) current curve for 120 min. DATS@MION-PEG-LF (10 µg/mL) with GSH (2 mM) at pH of 7.4, 6.5, and 8.0 (37 °C), and DATS@MION-PEG-LF (10 µg/mL) with GSH (2 mM) at 37, 20, and 4 °C (pH of 7.4) were also evaluated for H_2_S release.

### Protection effects of DATS@MION-PEG-LF from hypoxia induced damage in rat cardiomyocytes and neurons

DATS@MION-PEG-LF (1 ~ 10 µg/mL) + GSH (2 mM) and saline of same volume (Control) were separately added to medium of cultured cardiomyocytes or neurons (n = 3). After incubation for 4 h, medium was removed and replaced with DMEM/F-12 without glucose and serum. Then cardiomyocytes/neurons were exposed to hypoxia (94 % N_2_, 5 % CO_2_, 1 % O_2_) for 4 h in a CO_2_ incubator (Forma SERIES II WATER JACKET, Thermo Scientific, MA, USA), followed by reoxygenation (5 % CO_2_) for 1 h. After which, the cell viability was evaluated by CCK-8 assay and compared with the Control.

After the hypoxia/reoxygenation procedure, lactate dehydrogenase (LDH) activities of each group were also measured to evaluate cytotoxicity using an assay kit (JianCheng, Nanjing, China) according to the manufacturer’s instructions. The absorbance was determined by a micro plate reader at 440 nm. The levels of cell apoptosis were also determined by flow cytometry by previous method [27].

### Biodegradation and biodistributon assessment of DATS@MION-PEG-LF

Like almost all nanoparticles, intravenously administered MIONs are eventually cleared by mononuclear phagocytic system such as liver [28]. To investigate the *in vivo* metabolism and biodegradation of DATS@MION-PEG-LF, BALB/c-nu mice (15 g, 4 w, n = 6) were injected via the tail vein with 10 mg/kg of the DATS@MION-PEG-LF nanoparticles. Six mice were injected with same volume of saline and underwent same procedures as control. *In vivo* MRI scan was performed on 3-T MRI scanner (Discovery MRI 750, GE Medical Systems, Milwaukee, WI, USA) with an animal 8-channel phased-array coil with the following parameters: FSE T2 Fat Suppress, slice thickness: 1.0 mm, spacing: 0.1 mm, TR: 4167.0 ms, TE: 68.0 ms, refocus flip angle: 142°, echo train length: 24, and bandwidth: 31.25 kHz. T2-weighted MRI was used to detect the aggregation of nanoparticles in mouse livers by evaluating the calculated signal intensity. The signal intensities of nanoparticles at 6, 12, 24 h, 2, 4, 6, 8, 10, 12, and 14 days after injection were obtained in a region of interest (liver region) placed at the fixed site on matched slices. Signal to noise ratio (SNR) were calculated to compare the relative signal intensity. Repeat the protocols, and the SNRs of iron nanoparticles in mouse brains and hearts were evaluated within 48 h to assess the biodistribution of nanoparticles in brain and heart.

### In vivo toxicity assessment of DATS@MION-PEG-LF

Sprague-Dawley rats were injected with DATS@MION-PEG-LF (10 mg/kg) through tail vein (n = 6). Rats were euthanized at 24 h, 7, 14, and 30 days after injection, and brain, heart, liver, spleen, lung and kidney organs were harvested and fixed in 4 % paraformaldehyde for 24 h at room temperature then embedded in paraffin and cut into 7 µm-thick slices. The sections were stained with H&E and imaged under a light microscope at × 40 magnification. Six high-power fields were randomly selected in each photograph. Hematological and serological examinations at 24 h, 7, and 30 days after injections of DATS@MION-PEG-LF (10 mg/kg) or same volume of saline (Control) were also evaluated (Additional file 1).

### Effect of DATS@MION-PEG-LF on heart rates and blood pressure

Sprague-Dawley rats (n = 6) were anesthetized with medetomidine hydrochloride (Domitor, 250 µg/kg, IP.) and ketamine hydrochloride (Ketamine, 50 mg/kg, IP.). Carotid arteries were cannulated and connected to Model SMUP-E4 Bioelectric Signals Processing System (MFLab301) to display rat real time heart rates and blood pressure, which were recorded every 1 h. DATS@MION-PEG-LF (10 mg/kg) or same volume of saline (Sham) was administrated by tail vein injection. Then change of blood pressure and heart rates was evaluated.

### Heart/Brain targeting assessment of DATS@MION-PEG-LF

DiR was loaded to the MION-PEG-LF and MION frameworks. The protocols of loading and the identification of DiR@MION-PEG-LF and DiR@MION were described in the Additional file 1. Immunofluorescence method was applied to study the *in vivo* targeting ability of DATS@MION-PEG-LF to brain and heart. Sprague-Dawley rats were injected with either DiR@MION-PEG-LF (10 mg/kg) or DiR@MION (10 mg/kg) by tail vein. At 24 h after injection, rats were anaesthetized and sacrificed to obtain the brain and heart tissues. The brain and heart tissues were dewaxed and hydrated in dimethylbenzene and a graded series of alcohol, frozen and embedded in Tissue-tek O.C.T., and sliced (10 µm). Cardiomyocytes cytoplasm were stained with wheat germ agglutinin (WGA, 1:100), neuron cytomembranes were stained with neurofilament protein NF200 (1:100) and goat anti-mouse IgG (1:200), and DAPI was used for labeling of nuclei. The slides were then mounted with an antifade medium and observed with a DMI 4000 fluorescence microscope (Leica Camera Co., Wetzlar, Germany) (detailed in Additional file 1).

### In vivo H_2_S release of DATS@MION-PEG-LF

Sprague-Dawley rats were anesthetized with medetomidine hydrochloride (250 µg/kg, *I.P.*) and ketamine hydrochloride (50 mg/kg, *I.P*.) (n = 6). Carotid arteries were cannulated for blood withdrawal. 5, 10 or 20 mg/kg of DATS@MION-PEG-LF were separately administrated by tail vein injection. Blood (0.1 mL) was withdrawn at time intervals (0–12 h) after administration. The blood collected was anticoagulated with heparin sodium (50 U/mL) and centrifuged (3000 rpm, 15 min) to obtain plasma. As to the H_2_S concentration of DATS@MION-PEG-LF in plasma cannot be assessed by real time H_2_S-selective microelectrode assay, therefore it was measured by HPLC method by an Agilent Technologies HPLC (1260 infinity, CA, USA) with fluorescence detection (λex: 390 nm and λem: 475 nm) and an Eclipse XDB-C18 column (150 ⋅ 4.6 mm, 5 µm) within 24 h, which was previously described [7, 29].

Repeat the experiments, and Sprague-Dawley rats (n = 6) administrated by DATS@MION-PEG-LF (10 mg/kg) or same volume of saline (Vehicle group) were sacrificed at 24 h after injection. Myocardium and cortices tissues were quickly acquired and stored at − 80 °C. Then small pieces of tissues (about 50 mg) were homogenated in 500 µL of PBS (pH 7.4). H_2_S concentration of the homogenate was determined by HPLC analysis described above. The protein content of tissue was measured by the bicinchoninic acid (BCA) method using a BCA Protein Assay Kit (Pierce, IL, USA). The H_2_S content of samples were quantified by protein content.

### Rat CA/CPR model

Sprague-Dawley rats were anesthetized and heparinized (0.2 mL, *I.P*.). Then the CA/CPR rat model was established by the previous method [30] (described in Additional file 1). Rats were randomized into three groups: (1) sham (n = 6), which were subjected to the same procedure as the other groups except for CA/CPR; (2) Control group (n = 6), which underwent 5 min of CA and received injection of same volume of saline; (3) DATS@MION-PEG-LF group (n = 6), which underwent 5 min of CA and received injection of DATS@MION-PEG-LF (10 mg/kg). Drugs were immediately injected after successful resuscitation. At 24 h after reperfusion, the myocardium and cortices tissues were quickly acquired and stored for subsequent experimental analysis.

### Quantitative assessment of neutrophil accumulation

Heart and brain tissues were assessed for the myeloperoxidase (MPO) activity as a marker of neutrophil accumulation. Tissues were homogenized in a solution containing 0.5% hexadecyltrimethylammonium bromide dissolved in 10 mM K_3_PO_4_ buffer (pH 7.0) and centrifuged for 30 min (20,000 *g*, 4 °C). Supernatant was allowed to react with tetramethylbenzidine (1.6 mM) and 0.1 mM H_2_O_2_, and the change in absorbance was measured by spectrophotometry at 650 nm. The MPO activity was defined as the quantity of enzyme degrading 1 mmol of hydrogen peroxide per min at 37 °C and expressed in milliunits per milligram protein.

### Antioxidant enzyme activities

A total of 50 mg heart or brain tissue was homogenized in a 50 mM ice-cold potassium phosphate buffer (pH 6.8). Superoxide dismutase (SOD) activity, catalase (CAT) activity and the malonydialdehyde (MDA) levels were determined by the previously reported method [8]. The activity of SOD and CAT, and the levels of MDA were all standardized by protein content, determined using a bicinchoninic acid (BCA) protein assay kit (Beytime Institute of Biotechnology, Nantong, China).

### Transferase‐mediated dUTP nick‐end labeling (TUNEL) assay

Acquired heart and brain tissues were stained with H&E, followed by a TUNEL assay: the cell nuclei were stained with 4′,6′-diamidino-2-phenylindole hydrochloride (DAPI) color development kits (Roche, Basil, CH) in accordance with the manufacturers’ instructions. The cell nuclei that stained green were defined as TUNEL-positive nuclei and were monitored using a Nikon invert fluorescence microscope. The proportion of TUNEL positive nuclei per 500 nuclei was quantified at a 200⋅ magnification.

### Western blot assay

A piece of ischemic brain or heart tissue was homogenized by a rotor-stator homogenizer in ice-cold RIPA buffer (Pierce, Pittsburgh, PA, USA), and incubated at 4 °C overnight. After boiling with loading buffer (Fermentas, Glen Burnie, MD, USA), denatured proteins were separated in SDS PAGE gel, and transferred onto PVDF membrane. The membrane was blocked with 5% nonfat milk, followed by incubation with primary antibody of Bcl-2, BAX and Caspase-3 (Abcam, Cambridge, MA, USA) at 4 °C overnight. HRP-conjugated secondary antibody (Kangchen Bio-tech, Beijing, China) was used to incubate the membrane for another 2 h. SuperSignal West Pico Chemiluminescent Substrate (Pierce, Pittsburgh, PA, USA) was poured on the membrane to develop the band captured by FluorChem Image System (Alpha Innotech, Santa Clara, CA, USA).

### Evaluation of heart and cerebral function

Repeated CA/CPR protocol (n = 6), and cardiac and cerebral function were evaluated at 24 h after CPR and drug injection. Cerebral function was evaluated by neurological deficit scale (NDS) and balance beam test as to the previous methods [31, 32]. After which, rats were anesthetized for transthoracic echocardiography assessment using the Philips IE 33 system and a 12–4 MHz linear transducer (S12-4, Philips, AMS, NED). Stroke volume, ejection fraction (EF) and fractional shortening (FS) were derived to evaluate cardiac function, which were performed by skilled observer blindly. Repeated the CA/CPR protocols and kept the rats for 30 days, and survival of the rats were compared between the DATS@MION-PEG-LF and Vehicle groups (n = 30).

### Statistical analysis

All statistics were performed using SPSS Statistics Base 17.0 for Windows. Continuous data were expressed as mean ± standard errors (SEM). One-way analysis of variance (ANOVA) was used to examine statistical comparisons between groups. The significant difference between two groups was analyzed by Student’s t test. Survival condition was analyzed using the Kaplan–Meier method. A value of P < 0.05 was considered to be significant. All authors had full access to, and take full responsibility for the integrity of the data.

## Supplementary information

**Additional file 1.** Additional information.

## Data Availability

All data generated or analyzed during this study are included in this published article.

## References

[1] Callans DJ (2019). Out-of-hospital cardiac arrest–the solution is shocking. N Engl J Med.

[2] Alan S, Go D, Mozaffarian VL, Roger EJ, Benjamin JD, Berry MJ, Blaha, et al. Heart disease and stroke statistics–2014 update: a report from the American Heart Association. Circulation. 2014; e28–92. 10.1161/01.cir.0000441139.02102.80 PMC5408159 24352519

[3] Albert CM, Chae CU, Grodstein F, Rose LM, Rexrode KM, Ruskin JN (2003). Prospective study of sudden cardiac death among women in the United States. Circulation..

[4] Silverman MG, Scirica BM (2016). Cardiac arrest and therapeutic hypothermia. Trends Cardiovasc Med.

[5] Vandiver M, Snyder SH (2012). Hydrogen sulfide: a gasotransmitter of clinical relevance. J Mol Med (Berl)..

[6] Powell CR, Dillon KM, Matson JB (2018). A review of hydrogen sulfide (H(2)S) donors: chemistry and potential therapeutic applications. Biochem Pharmacol..

[7] Sun X, Wang W, Dai J, Huang J, Shi M, Chu X (2018). Donor heart preservation with a novel long-term and slow-releasing hydrogen sulfide system. Nitric Oxide..

[8] Sun X, Wang W, Dai J, Jin S, Huang J, Guo C (2017). A long-term and slow-releasing hydrogen sulfide donor protects against myocardial ischemia/reperfusion injury. Sci Rep.

[9] Li L, Whiteman M, Guan YY, Neo KL, Cheng Y, Lee SW (2008). Characterization of a novel, water-soluble hydrogen sulfide-releasing molecule (GYY4137): new insights into the biology of hydrogen sulfide. Circulation..

[10] Xiaotian S, Kong B, Wang W, Chandran P, Selomulya C, Zhang H (2015). Mesoporous silica nanoparticles for glutathione-triggered long-range and stable release of hydrogen sulfide. J Mater Chem B.

[11] Bharti C, Nagaich U, Pal AK, Gulati N (2015). Mesoporous silica nanoparticles in target drug delivery system: A review. Int J Pharm Investig..

[12] Wang W, Liu H, Lu Y, Wang X, Zhang B, Cong S (2019). Controlled-releasing hydrogen sulfide donor based on dual-modal iron oxide nanoparticles protects myocardial tissue from ischemia-reperfusion injury. Int J Nanomed.

[13] Chen L, Wu Y, Wu H, Li J, Xie J, Zang F (2019). Magnetic targeting combined with active targeting of dual-ligand iron oxide nanoprobes to promote the penetration depth in tumors for effective magnetic resonance imaging and hyperthermia. Acta Biomater..

[14] Geurts M (2014). Therapeutic hypothermia and the risk of infection: a systematic review and meta-analysis. Crit Care Med.

[15] Raynal I, Prigent P, Peyramaure S, Najid A, Rebuzzi C, Corot C (2004). Macrophage endocytosis of superparamagnetic iron oxide nanoparticles: mechanisms and comparison of ferumoxides and ferumoxtran-10. Invest Radiol.

[16] Semelka RC, Helmberger TK (2001). Contrast agents for MR imaging of the liver. Radiology..

[17] Huang G, Zhang C, Li S, Khemtong C, Yang S-G, Tian R (2009). A Novel Strategy for Surface Modification of Superparamagnetic Iron Oxide Nanoparticles for Lung Cancer Imaging. J Mater Chem..

[18] Banerjee SS, Aher N, Patil R, Khandare J (2012). Poly(ethylene glycol)-prodrug conjugates: concept, design, and applications. J Drug Deliv.

[19] Huang R, Ke W, Han L, Liu Y, Shao K, Ye L (2009). Brain-targeting mechanisms of lactoferrin-modified DNA-loaded nanoparticles J Cereb Blood Flow Metab..

[20] Hu K, Shi Y, Jiang W, Han J, Huang S, Jiang X (2011). Lactoferrin conjugated PEG-PLGA nanoparticles for brain delivery: preparation, characterization and efficacy in Parkinson’s disease. Int J Pharm.

[21] Dauber IM, VanBenthuysen KM, McMurtry IF, Wheeler GS, Lesnefsky EJ, Horwitz LD, Weil JV (1990). Functional coronary microvascular injury evident as increased permeability due to brief ischemia and reperfusion. Circ Res.

[22] Paulis LE, Geelen T, Kuhlmann MT, Coolen BF, Schäfers M, Nicolay K (2012). Distribution of lipid-based nanoparticles to infarcted myocardium with potential application for MRI-monitored drug delivery. J Control Release.

[23] Sodha NR, Clements RT, Feng J, Liu Y, Bianchi C, Horvath EM (2008). The effects of therapeutic sulfide on myocardial apoptosis in response to ischemia-reperfusion injury. Eur J Cardiothorac Surg.

[24] Wang W, Sun X, Zhang H, Yang C, Liu Y, Yang W (2016). Controlled release hydrogen sulfide delivery system based on mesoporous silica nanoparticles protects graft endothelium from ischemia-reperfusion injury. Int J Nanomed..

[25] Corine C, Visser L, Heleen Voorwinden LR, Harders M, Eloualid L, van Bloois, Daan JA, Crommelin (2004). Coupling of metal containing homing devices to liposomes via a maleimide linker: use of TCEP to stabilize thiol-groups without scavenging metals. J Drug Target.

[26] Katebi S, Esmaeili A, Ghaedi K, Zarrabi A (2019). Superparamagnetic iron oxide nanoparticles combined with NGF and quercetin promote neuronal branching morphogenesis of PC12 cells. Int J Nanomed.

[27] Jiang L, Zhong J, Dou X, Cheng C, Huang Z, Sun X (2015). Effects of ApoE on intracellular calcium levels and apoptosis of neurons after mechanical injury. Neuroscience..

[28] Kiessling F, Mertens ME, Grimm J, Lammers T (2014). Nanoparticles for imaging: top or flop?. Radiology.

[29] Tan B, Jin S, Sun J, Gu Z, Zhu X, Sun Y (2017). New method for quantification of gasotransmitter hydrogen sulfide in biological matrices by LC-MS/MS. Sci Rep..

[30] Qin S, Chen M-H, Fang W, Tan X-F, Lu X, Yang Y-G (2019). Cerebral protection of epigallocatechin gallate (EGCG) via preservation of mitochondrial function and ERK inhibition in a rat resuscitation model. Drug Des Devel Ther.

[31] Jia X, Koenig MA, Shin H-C, Zhen G, Pardo CA, Hanley DF (2008). Improving neurological outcomes post-cardiac arrest in a rat model: immediate hypothermia and quantitative EEG monitoring. Resuscitation..

[32] Hausser N, Johnson K, Parsley MA, Guptarak J, Spratt H, Sell SL (2018). Detecting behavioral deficits in rats after traumatic brain injury. J Vis Exp..

