# Supplementary material for: Novel controlled and targeted releasing hydrogen sulfide system exerts combinational cerebral and myocardial protection after cardiac arrest
Source: J Nanobiotechnology. 2021 Feb 6;19:40. doi: 10.1186/s12951-021-00784-w (PMC7866762; doi:10.1186/s12951-021-00784-w)
Supplement: Supplementary file 1 — Additional file 1. Additional information. [file 12951_2021_784_MOESM1_ESM.docx]

***Additional Information***

**Novel Controlled and Targeted Releasing Hydrogen Sulfide System Exerts Combinational Cerebral and Myocardial Protection after Cardiac Arrest**

Xiaotian Sun^1†^*, Yiqing Wang^†1^，Shuyan Wen^1^, Kai Huang^1^, Jiechun Huang^1^, Xianglin Chu^1^, Fangrui Wang^1^, Liewen Pang^1^

1: From the Departments of Cardiothoracic Surgery, Huashan Hospital of Fudan University, Shanghai, China.

† These authors contributed equally to this work.

***Corresponding author**:

Xiaotian Sun, MD, PhD, 12^th^ Wulumuqi Rd, Shanghai, 200040, China (E-mail: [sunxiaotian@126.com](mailto:sunxiaotian@126.com))

**Funding:** This work was supported by the National Natural Science Foundation of China (81601663, 81772042), Shanghai Shen Kang Clinical Research Cultivation Project (SHDC12018X18), and Natural Science Foundation of Shanghai (19ZR1407300).

**Conflict of Interest Disclosures**: All authors declare no conflict of interest in this work.

**Keyword**: Cardiac arrest; Hydrogen sulfide; Mesoporous iron oxide nanoparticles; Ischemia and reperfusion injury; Combinational cerebral and myocardial protection.

***
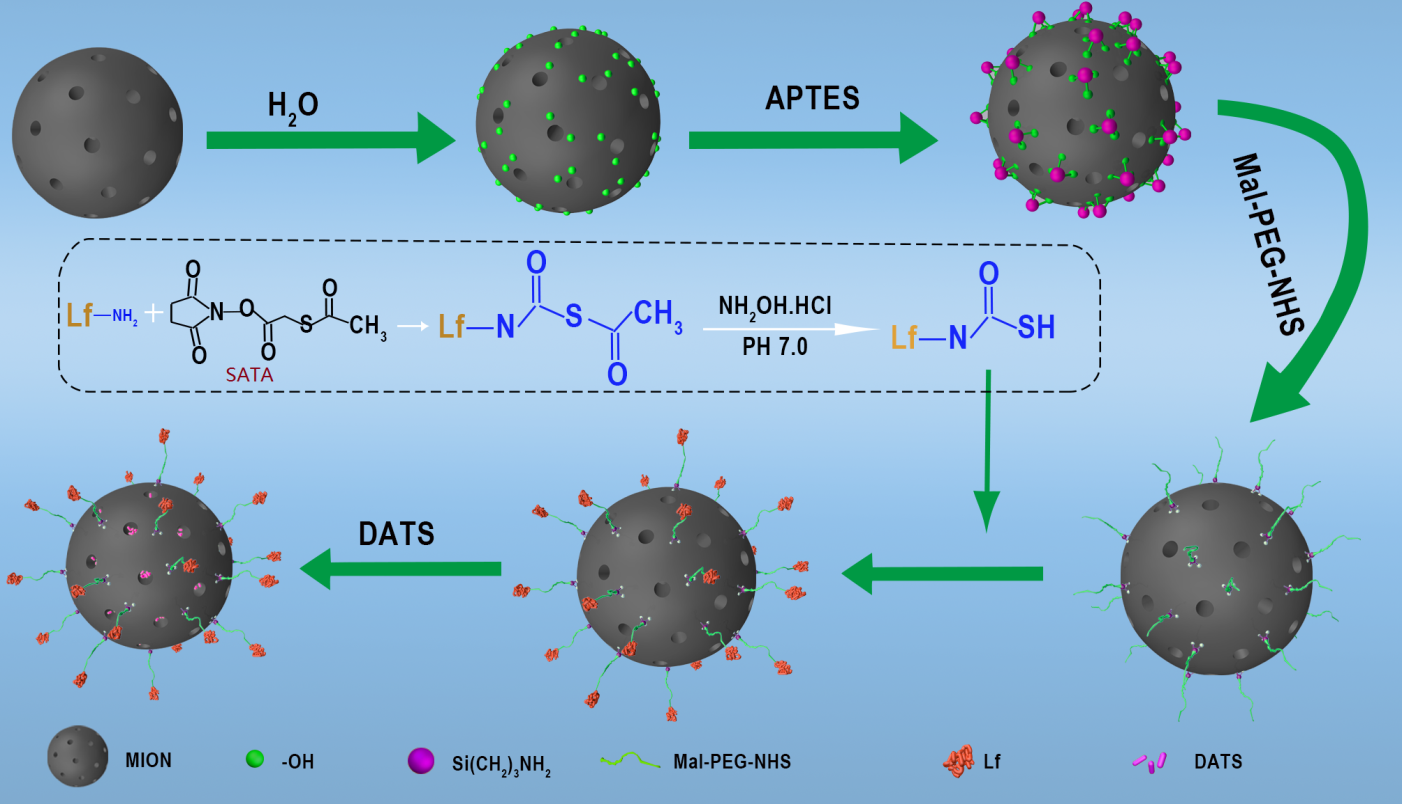
***

**Figure S1**. Procedures of modification and drug loading of MIONs.

***FT-IR Spectroscopy Assessment***

The conjugation of Mal-PEG-NHS and LF to MIONs were confirmed by FT-IR spectroscopy (Perkin Elmer Frontier FT-IR). FT-IR spectra of freeze-dried samples were recorded from wavenumber 400-4000 cm^-1^. As shown in **Supplementary** **Figure. 2a**, the bands at 650 cm^-1^ different between Line A and Line B confirmed the typical bond of C-S in MION-PEG-LF, which is the evidence of the conjugation of thiolate LF. Comparing the spectra of MIONs (**Supplementary** **Figure. 2b**) and MION-PEG-LF (**Supplementary** **Figure. 2c**) over the wavenumber of 400~4000 cm^-1^, the strong peak of MION-PEG-LF at 1100 cm^-1^ was generally attributable by the C-O vibration in alcohol molecules of PEG (**Supplementary** **Figure. 2d**).

***
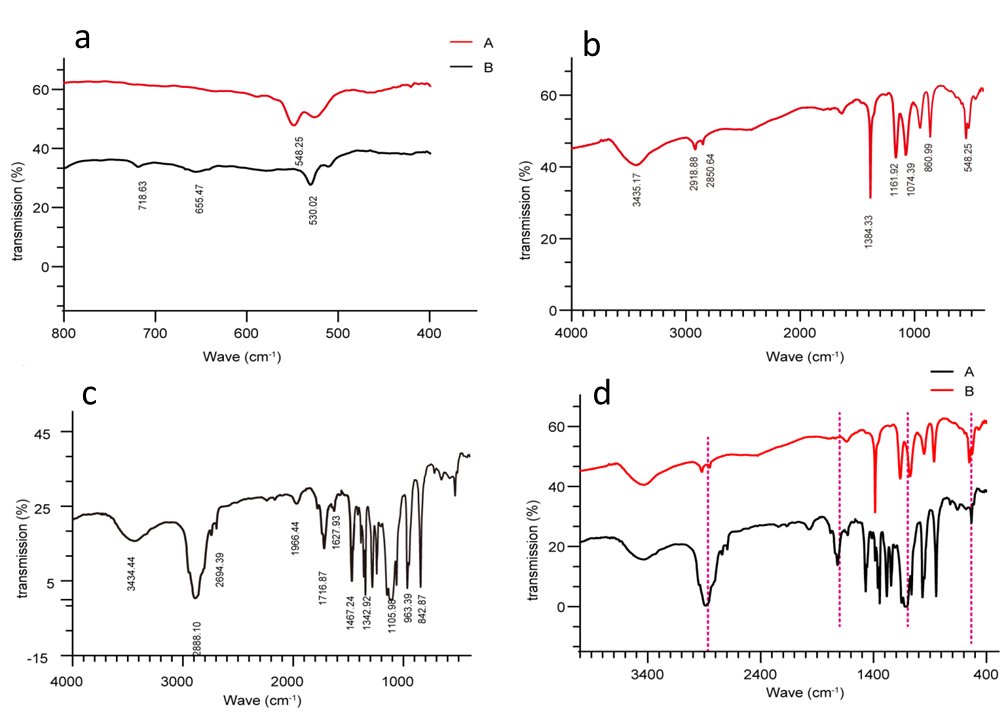
***

**Figure S2.** FT-IR Spectroscopy Assessment for DATS@MION-PEG-LF.

***Culture of neonatal cardiomyocytes and cortical neurons***

Primary neonatal cardiomyocytes and cortical neurons were obtained from myocardium and the cerebral cortices tissues of newborn Sprague-Dawley rats (6 g, 24 h) Isolated cardiomyocytes were seeded at a density of 1×10^5^ cells/mL in Dulbecco's Modified Eagle's Medium/F12 (DMEM/F12, Gibco Invitrogen, CA, USA) supplemented with 10 % fetal bovine serum, 100 U/mL penicillin, 100 mg/mL streptomycin, and 100 mM 5-bromodeoxyuridine. Cells were cultured in a humidified incubator at 37 °C with 95 % air and 5% CO_2_ for 72 h. Neuron suspensions were diluted and plated at density of 7×10^4^/ 1.13 cm^2^ on sterile 12 mm diameter round glass coverslips (VWR®, Leuven, Belgium) pre-coated with polylysine in 24-well dishes (Greiner Bio-One, Vilvoorde, Belgium). Cultures were placed in an incubator at 37 °C with humid atmosphere at 5 % CO_2_. Cells were fed with fresh medium (1% FBS) twice per week.

***Protocols of loading and identification of DiR into the MION or MION-PEG-LF***

Dialkylcarbocyanine (DiR) is a near-infrared (NIR) fluorescent dye commonly used in organic nanocarriers like poly-meric micelles, liposomes and exosomes, however, it could also be loaded into MIONs due to the inner hydrophobic environment of the latter (Wang, Biomaterials 75: 71-81, 2016). The protocols of DiR loaded into the MION or MION-PEG-LF frameworks were as following: 1 mg of DiR was dissolved in 0.5 mL DMSO, and then 5 mL of MION or MION-PEG-LF (1mg/mL) was added. After stirring for 4 h at room temperature, the DiR@MION-PEG-LF or DiR@MION complex was washed with PBS thrice, followed by centrifuging for 30 min at 12000 rpm and dialyzed in PBS for 48 h to remove the free DiR. The successfully loading of DiR to MION-PEG-LF or MION was confirmed by the *ex vivo* imaging by Bruker Xtreme system (Xtreme system, CA). Briefly, 0.1 mL of MION (10mg/mL), DiR@MION-PEG-LF (10mg/mL), DiR@MION (10mg/mL), and free DiR (1mg/mL) were separately added to a 96-well plate; then the plate was placed into the Xtreme system in the ventral position, followed by 30 s of exposure for X-ray and 30 s of exposure for fluorescence (ex: 710 nm, em: 760 nm).

As shown in **Supplementary** **Figure. 3**, the isolated solutions of DiR@MION and DiR@MION-PEG-LF both showed steady and obvious fluorescence signals compared with MIONs and free DiR, proving that the fluorescent signals of DiR@MION and DiR@MION-PEG-LF were able to represent the distributions of themselves in the *in vivo* experiments.

***
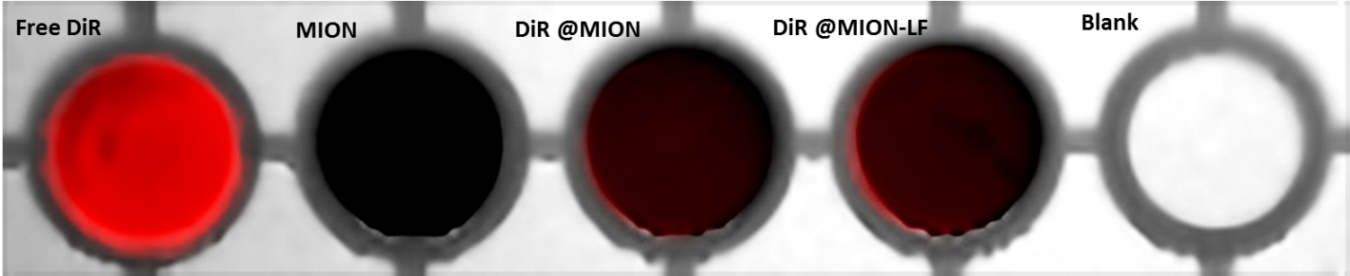
***

**Figure S3.** *Ex vivo* imaging of DiR@MION, DiR@MION-PEG-LF, MION, and free DiR.

***Heart Immunofluorescence Study***

The paraffin sections were dewaxed and hydrated in dimethylbenzene and a graded series of alcohol. The slides were then retrieved by citric acid buffer (PH of 6.0) microwave antigen retrieval. After microwave irradiation, the myocardial tissues were rinsed with three changes of PBS (pH 7.4) for 5 min each time. For staining, the samples were firstly covered with wheat germ agglutinin (WGA) solution in a dark chamber at 37°C. After 30 min the WGA solution was removed and the slides were rinsed again with three changes of PBS (pH 7.4) for 5 minutes each time. For labeling of nuclei, 4',6-diamidino-2-phenylindole (DAPI) was used. The samples were covered with DAPI solution in a dark chamber at room temperature. After 10 min the DAPI solution was removed and the slides were rinsed with PBS in the same way. The slides were finally mounted with an antifade medium and observed with a DMI 4000 ﬂuorescence microscope (Leica Camera Co., Wetzlar, Germany) (The excitation wavelength of DAPI was 466 nm and the emission wavelength was 504 nm. The excitation wavelength of DiR was 750 nm and the emission wavelength was 782 nm). The DAPI-stained nuclei gave out blue fluorescence under excitation and DiR-labeled nanoparticles showed red fluorescence under excitation.

***Brain Immunofluorescence Study***

The paraffin sections were dewaxed and hydrated in dimethylbenzene and a graded series of alcohol. The slides were then retrieved by citric acid buffer (PH of 6.0) microwave antigen retrieval. After microwave irradiation, the brain tissues were rinsed with three changes of PBS (pH 7.4) for 5 min each time. The samples were then incubated with 3 % bovine serum albumin (BSA) for 30 min at room temperature. BSA was removed before staining. The samples were firstly covered with the solution of neurofilament protein NF200 (1: 100) in a wet box at 4°C. After overnight incubation, the slides were rinsed with three changes of PBS (pH 7.4) for 5 min each time. Goat anti-mouse IgG (1:200) were then added to the samples. After 50 min of incubation in a dark chamber at room temperature, the slides were rinsed again with three changes of PBS (pH 7.4) for 5 min each time. For labeling of nuclei, DAPI was used. The samples were covered with DAPI solution in a dark chamber at room temperature. After 10 min the DAPI solution was removed and the slides were rinsed with PBS in the same way. The slides were finally mounted with an antifade medium and observed with a DMI 4000 ﬂuorescence microscope (Leica Camera Co., Wetzlar, Germany) (The excitation wavelength of DAPI was 466 nm and the emission wavelength was 504 nm. The excitation wavelength of DiR was 750 nm and the emission wavelength was 782 nm). The DAPI-stained nuclei gave out blue fluorescence under excitation and DiR-labeled nanoparticles showed red fluorescence under excitation.

***Cardiac arrest /*** ***cardiopulmonary resuscitation model***

Rats were applied left femoral artery intubation with PE-50 silica gel tubes (Becton-Dickinson, Franklin Lakes, NJ, USA) filled with 5 IU/mL heparin sodium solution. A pressure transducer was connected with the femoral artery lien tube for blood pressure monitoring. Three needle electrodes were placed subcutaneously on both upper limbs and the right lower limb respectively, and the standard II lead electrocardiogram was recorded. All the data was obtained by BL-420F biological system (Chengdu Taimeng Instrument Co. Ltd.) After the arterial pressure and electrocardiogram were stabilized for 5 min, the cardiac arrest (CA) was induced by transoesophageal electrical induction. Briefly, CA was defined as ventricular fibrillation or electromechanical dissociation with a mean aortic pressure (MAP) ≤20 mmHg. After 5 min of CA, cardiopulmonary resuscitation (CPR) including manual chest compression and mechanical ventilation with air was started. After 1 min of CPR, one dose of epinephrine (20 μg/kg) was given through a left femoral vein catheter. Restoration of spontaneous circulation (ROSC) was defined as an organized cardiac rhythm with a mean aortic pressure (MAP) >60 mmHg for ≥1 min. When there was a failure of ROSC after 5 min, resuscitation efforts were discontinued. From anesthesia to awakening, the rectal temperature of the rats was monitored continuously and maintained by a heating lamp at 37.0 ± 0.5 °C.

***Hematological and serological examinations***

At 2 h, 24 h and 7 d after injection of DATS@MION-PEG-LF (10 mg/kg) and saline (Control) into the rats (n = 6), blood was collected for hematological analysis and evaluation of hepatic and renal function. For hematological analysis, blood was preserved in sodium ethylenediaminetetra-acetic acid tubes. The red blood cells (RBC), white blood cells (WBC), platelets (PLT), the hemoglobin level (HGB), hematocrit (HCT) and white blood cell distribution (neutrophils, lymphocytes) were all determined by Automatic Hematological Analyzer (XE-2100, SYSMEX, Japan). The levels of aspartate aminotransferase (AST), alanine aminotransferase (ALT), serum creatinine (CRE) and urea nitrogen in the serum (BUN) were determined using Automatic Analyzer (7600-120, HITACHI, Japan) according to the instruction provided by the commercial assay kits.

**Table S1: Hematological and serological results of rats treated with DATS@MION-PEG-LF**

|  | **Control** | **24 h** | **7 d** | **30 d** |
| --- | --- | --- | --- | --- |
| **WBC [K μL^-1^]**  **NEU %**  **LY %** | 6.3 ± 1.23  7.39 ± 2.82  93.5 ± 7.9 | 6.47 ± 1.45  7.31 ± 2.93  91.3 ± 6.5 | 6.53 ± 2.02  7.01 ± 2.56  89.3 ± 8.2 | 6.448 ± 2.13  6.93 ± 2.42  95.1 ± 9.1 |
| **RBC [M μL^-1^]** | 6.45 ± 0.82 | 6.72 ± 0.53 | 6.92 ± 0.61 | 7.03 ± 0.63 |
| **HGB [g dL^-1^]** | 143.3 ± 11.2 | 138.2 ± 12.7 | 153.0 ± 8.8 | 145.2 ± 10.9 |
| **HCT %** | 44.2 ± 3.6 | 47.3 ± 2.9 | 43.8 ± 3.8 | 42.9 ± 4.0 |
| **PLT [K μL^-1^]** | 902.7 ± 87.4 | 912.5 ± 90.5 | 932.8 ± 67.1 | 923.7 ± 92.6 |
| **ALT [U L^-1^]** | 35.2 ± 6.1 | 37.5 ± 5.2 | 33.2 ± 4.6 | 35.6 ± 3.0 |
| **AST [U L^-1^]** | 89.4 ± 7.5 | 90.3 ± 9.6 | 92.5 ± 8.9 | 93.8 ± 8.7 |
| **BUN [mg dL^-1^]** | 6.13 ± 0.99 | 6.54 ± 1.01 | 6.02 ± 0.65 | 5.98 ± 1.11 |
| **CRE [mg dL^-1^]** | 15.9 ± 3.2 | 16.2 ± 2.2 | 17.5 ± 3.3 | 16.8 ± 3.5 |

Data were presented as mean ± SEM (n = 6) .There was no significant difference of any data among groups. WBC: white blood cells, NEU: neutrophils, LY: lymphocytes, RBC: red blood cells, HGB: hemoglobin level, HCT: hematocrit, PLT: platelets, ALT: alanine aminotransferase, AST: aspartate aminotransferase, BUN: urea nitrogen and CRE: serum creatinine.

***In vivo Pharmacokinetics Study***

The pharmacokinetics behavior of DATS@MION-PEG-LF was studied in rats through the tail vein injection of different doses of DATS@MION-PEG-LF (5 mg/kg, 10 mg/kg and 20 mg/kg). The pharmacokinetic parameters of the different dose groups were presented in **Supplementary Table. 2,** presenting that DATS@MION-PEG-LF stably and continuously release H_2_S in plasma.

**Table S2. Pharmacokinetic parameters of DATS@MION-PEG-LF after different dosage intravenous to rat**

|  | **5 mg** | **10 mg** | **20 mg** |
| --- | --- | --- | --- |
| **t_1/2_ (h)**  **Kel (h^-1^)**  **CL (mg/kg)/(umol/L)/h** | 56.37±9.09  0.013±0.002  0.070±0.011 | 50.43±19.00 0.015±0.004  0.143±0.030 | 64.22±32.23  0.013±0.004  0.236±0.067 |
| **MRT_0-t_ (h)** | 11.577±0.108 | 11.582±0.069 | 11.621±0.107 |
| **MRT_0→∞_ (h)** | 82.36±12.49 | 74.02±26.51 | 93.48±45.58 |
| **AUC_0→t_ (umol·h/L)** | 17.88±0.42 | 19.92±0.33 | 21.31±0.58 |
| **AUC_0→∞_ (umol·h/L)** | 72.99±9.96 | 73.87±20.18 | 94.58±35.02 |

Data were presented as mean ± SD (n = 6). t_1/2_: distribution half-life time; Kel: elimination rate constant; CL: the clearance rate; MRT: mean retention time (MRT), AUC: area under the curve.
